# Age-Related Decline in Gangliosides GM1 and GD1a in Non-CNS Tissues of Normal Mice: Implications for Peripheral Symptoms of Parkinson’s Disease

**DOI:** 10.3390/biomedicines11010209

**Published:** 2023-01-14

**Authors:** Suman Chowdhury, Gusheng Wu, Zi-Hua Lu, Ranjeet Kumar, Robert Ledeen

**Affiliations:** Department of Pharmacology, Physiology, and Neuroscience, Rutgers, The State University of New Jersey, Newark, NY 07103, USA

**Keywords:** Parkinson’s disease, GM1 ganglioside, aging, motor impairment, memory impairment, peripheral tissues, HPTLC

## Abstract

The purpose of this study was to determine whether the age-related decline in a-series gangliosides (especially GM1), shown to be a factor in the brain-related etiology of Parkinson’s disease (PD), also pertains to the peripheral nervous system (PNS) and aspects of PD unrelated to the central nervous system (CNS). Following Svennerholm’s demonstration of the age-dependent decline in a-series gangliosides (both GM1 and GD1a) in the human brain, we previously demonstrated a similar decline in the normal mouse brain. The present study seeks to determine whether a similar a-series decline occurs in the periphery of normal mice as a possible prelude to the non-CNS symptoms of PD. We used mice of increasing age to measure a-series gangliosides in three peripheral tissues closely associated with PD pathology. Employing high-performance thin-layer chromatography (HPTLC), we found a substantial decrease in both GM1 and GD1a in all three tissues from 191 days of age. Motor and cognitive dysfunction were also shown to worsen, as expected, in synchrony with the decrease in GM1. Based on the previously demonstrated parallel between mice and humans concerning age-related a-series ganglioside decline in the brain, we propose the present findings to suggest a similar a-series decline in human peripheral tissues as the primary contributor to non-CNS pathologies of PD. An onset of sporadic PD would thus be seen as occurring simultaneously throughout the brain and body, albeit at varying rates, in association with the decline in a-series gangliosides. This would obviate the need to postulate the transfer of aggregated α-synuclein between brain and body or to debate brain vs. body as the origin of PD.

## 1. Introduction

Parkinson’s disease (PD) is the most prevalent of the synucleinopathies, which are characterized by the accumulation of misfolded, aggregated alpha-synuclein (aSyn) in neurons of the central nervous system (CNS) and peripheral nervous system (PNS) [1]. That such Lewy accumulations characterize PD pathology in the PNS as well as the CNS, was well illustrated in the landmark studies of Braak and coworkers, among others, demonstrating the early involvement of the autonomic and olfactory nervous systems [2,3,4,5]. These occur prior to overt movement disorders in sporadic PD, which derive from progressive degeneration of dopaminergic (DA) neurons of the substantia nigra pars compacta (SNpc); later manifestations often involving cognitive dysfunctions are also featured in Braak’s staging hypothesis. This led to the proposal that PD originates in the enteric nervous system, with inclusions formed there migrating in a prion-like manner to the cortical motor nucleus of the vagus and intermediolateral cell columns of the sympathetic system into the brain [6,7]. This idea was supported by subsequent animal studies demonstrating such migration of αSyn aggregates [8,9]. 

Although Braak’s staging hypothesis has been criticized as inconsistent with some findings [10], it was useful in showing that certain non-motor symptoms of PD are well coordinated with neuropathological alterations outside of the SNpc [5]. However, other lines of evidence have indicated that at least some cases of sporadic PD do not have such indications including that of Lewy pathology in the dorsal motor nucleus of the vagus [11]; rather they show a limbic-predominant distribution of aSyn inclusions involving the SNpc [12,13]. This dichotomy triggered a debate on brain-first vs. body-first as the origin of PD leading to the proposed existence of two subtypes of PD [9,14]. 

Our findings on the systemic deficiency of GM1 and GD1a gangliosides in brain and peripheral tissues of PD patients, together with our proposal that such a deficiency is what impairs neuronal function and viability as an inducing cause of PD [15], have led us to propose an alternative interpretation of these disparate findings: that a progressive body-wide decline of GM1 leading to body-wide aggregation of αSyn in neurons of the CNS and PNS is the predominant cause of sporadic PD and occurs simultaneously in the neurons of the brain and the periphery. Hence it is unnecessary (and likely inaccurate) to postulate the prion-like transfer of aggregated aSyn between the brain and body. Such transfers likely do occur [16] but most probably on a limited scale considering the improbability of such transfer occurring to such diverse and distant sites as the gastrointestinal, cardiovascular, dermatological, SNpc, and other tissues manifesting PD symptoms. The present study provides evidence for the progressive, age-related decline of a-series gangliosides in the PNS of normal mice, analogous to that previously demonstrated for the CNS of such mice and also analogous to that believed to occur in the PNS of humans. We propose that such a reduction in a-series gangliosides (especially GM1), which is even more pronounced in PD, impairs neuronal function through well-delineated mechanisms including failed interaction with aSyn (see Discussion). 

## 2. Materials and Methods

### 2.1. Mouse Breeding

Mice (C57BL/6) obtained initially from the Jackson Labs were bred over several generations in our own breeding colony with maintenance in the Rutgers New Jersey Medical School Animal Facility. This employed 12 h light/dark cycles and all animal procedures were in accord with the guidelines of the Rutgers Institutional Animal Care and Use Committee (IACUC). Mice of both genders were employed at the ages indicated in each study.

### 2.2. Ganglioside Analysis

Mice were euthanized by cardiac perfusion with phosphate-buffered saline followed by removal of the desired tissues via dissection. The heart, colon, and skin tissues of WT mice at 60, 90, 191, 334, and 586 days of age (DOA) were cut into small pieces and extracted with chloroform (C) methanol (M) (1/1, by volume) employing 1.5 mL per 50 mg of tissue to remove total lipids, as described [17]. Two mice (one male and one female) were employed for HPTLC depiction and numerical quantification at each time point. An additional two mice (one male and one female) were employed for HPTLC depiction only (without quantification) for each time point (see Supplementary Materials). In brief, tissues were homogenized in this solvent using a thermofixer at 1400 rpm for 15 min. Proteins and other nonlipids were pelleted by centrifugation and the lipid-containing supernatant was removed, followed by an additional extraction using C/M (2/1 by vol). The combined solution of lipids was adjusted to C/M 2/1 (by vol) by the addition of chloroform, and the extracted lipids were separated into aqueous and organic phases by the addition of 20% water with thorough mixing. After settling into two clear phases, the upper methanol–water phase (containing the gangliosides) was evaporated to dryness with a nitrogen stream and the resulting solid was reconstituted in a small volume of C/M; measured amounts were applied to HPTLC plates that had been developed in C/M/0.2 M KCl (5/4/1). GM1 was detected with Cholera toxin B linked to horseradish peroxidase (CtxB-HRP), and the same was applied to the other ganglio-series gangliosides following their conversion to GM1 via the reaction of the plate with neuraminidase, as described [18,19]. GM1 and GD1a were quantified using ImageJ software. 

### 2.3. Motor and Memory Impairment Tests

Grip duration, irritant removal, and memory impairment tests were carried out as described in previous studies [20,21]. A total of 372 mice of both genders were employed from 21 to >501 DOA and studied for memory impairment, where each age group sample number varied (6–36 mice) depending on the availability. 

*Grip duration test:* An assessment of physical impairment was conducted by having the mouse cling with its forepaws to a narrow horizontal rod located 50 cm above a pillow and measuring the time taken to fall from the rod [21]. Grip duration tests were performed three consecutive times, with rest intervals of 30 min between each trial, and the readings were averaged. 

*Irritant removal test:* This is another physical test used to assess motor response to a sensory stimulus [22], also called the adhesive removal test. Each mouse was measured five times and the shortest three-time durations were averaged.

*T-Maze test:* The T-maze forced-trial spontaneous alternation test was conducted on mice to test for differences in spatial memory capacity [21]. Three trials are performed with a 1 h interval between each trial for each mouse. At the end of all trials, the total score was calculated per mouse and the correct rate was calculated and reported as a percentage of correct responses. Thesecorrect responses were called the % alternation rate. 

### 2.4. Statistical Analysis

Statistical comparisons for behavioral and memory tests were performed using one-way ANOVA plus Bonferroni’s multiple comparison tests. Data are shown as average ± SEM (each age group sample number varied depending on the availability). The number of HPTLC samples was not sufficient for statistical analysis (male and female separately) but we feel the HPTLC depictions indicate a strong reduction in a-series gangliosides with age.

## 3. Results

In the present study, we examined the effect of aging on the expression of a-series gangliosides in the peripheral tissues of normal wild-type (WT) mice. We focused on peripheral tissues since they are well documented as showing pathological involvement in PD including the colon, heart, and skin of mice between 60 to 586 DOA. Ganglioside analysis using HPTLC was employed for two male and two female samples from each age group. Representative HPTLC images are shown below (and also in Supplementary Materials Figures S1–S3). Additionally, we also performed behavioral tests to assess the motor and memory performance of such mice. A total of 372 mice (male and female combined) were studied, where each age group sample number varied (6 to 36) depending on their availability. From the study, it became evident that both male and female WT mice showed a similar profile of age-dependent differences in ganglioside distribution, which changed significantly with aging in peripheral tissues as well as in motor and memory impairment. 

### 3.1. Ganglioside Changes in Peripheral Tissues of Normal Mice with Aging

**Figure 1:**
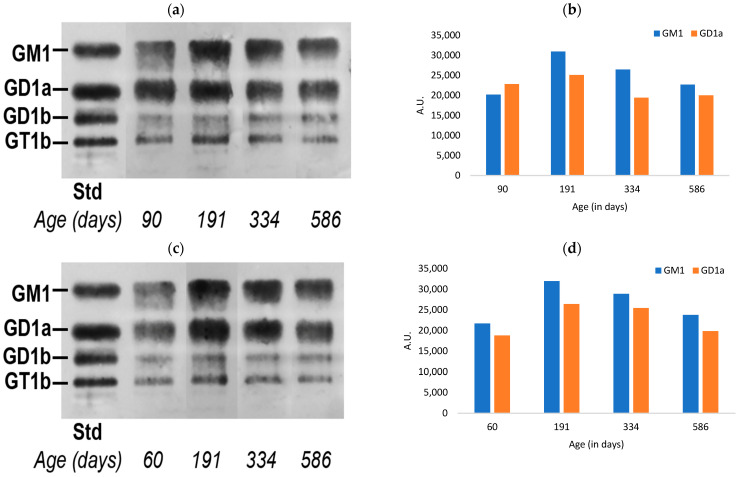
Quantification of a-series gangliosides in the colon tissue of aging mice: (**a**) HPTLC of male mice colon gangliosides; (**b**) relative quantitation of the intensity of GM1 and GD1a; (**c**) HPTLC of female mice colon gangliosides; and (**d**) relative quantitation of the intensity of GM1 and GD1a (A.U. = arbitrary units).

The C57BL/6 WT strain of mice showed age-dependent changes in the ganglioside content present in the colon (Figure 1), with both male and female mice showing an increase in both a-series gangliosides between 60 and 191 DOA followed by a marked decline in those between 191 and 586 DOA. There did appear, however, to be a modest gender difference with a slightly more rapid decline in males, as seen in comparing the decreases between 191 and 334 DOA. The other ganglio-series gangliosides (GD1b and GT1b) were much less prominent and were not quantified.

**Figure 2:**
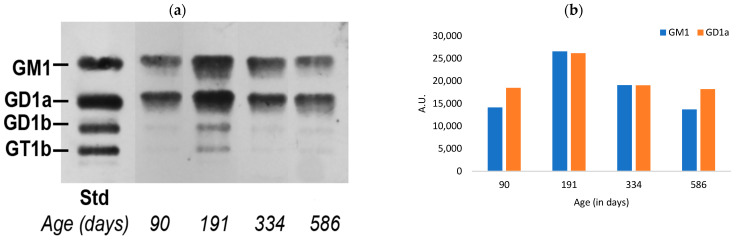

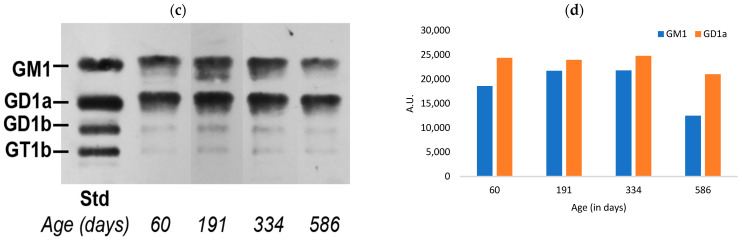
Quantification of a-series gangliosides in the skin tissue of aging mice: (**a**) HPTLC of male mice skin gangliosides; (**b**) relative quantitation of the intensity of GM1 and GD1a; (**c**) HPTLC of female mice skin gangliosides; (**d**) relative quantitation of the intensity of GM1 and GD1a (A.U. = arbitrary units).

Similar results were obtained for skin, while the gender difference noted for the colon was even more pronounced for this peripheral tissue; both of the a-series dropped sharply between 191 and 334 DOA for the male while showing virtually no decline for the female. However, both showed a prominent decline at 586 DOA (Figure 2). 

**Figure 3:**
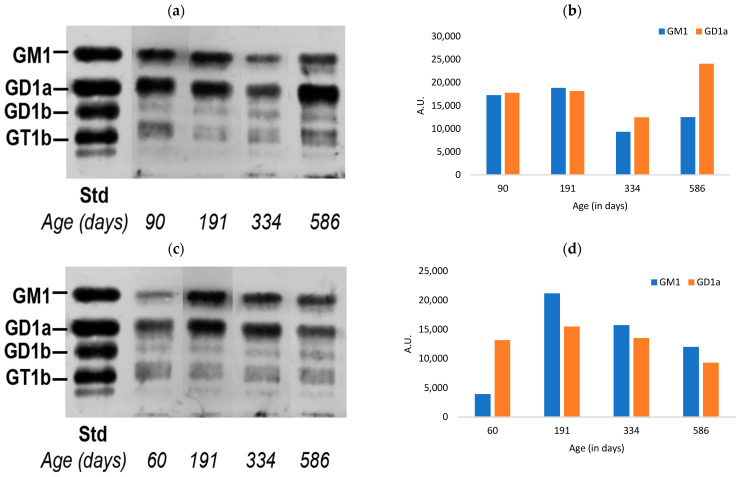
Quantification of a-series gangliosides in the heart tissue of aging mice: (**a**) HPTLC of male mice heart tissue for the detection of gangliosides; (**b**) relative quantitation of the intensity of gangliosides; (**c**) HPTLC of female mice heart tissue for the detection of GM1 and GD1a; (**d**) relative quantitation of the intensity of GM1 and GD1a (A.U. = arbitrary units).

Finally, our study of the heart yielded a generally similar pattern except for an apparent aberrant rise in the a-series of the male mouse at 586 DOA (Figure 3). This rise was seen despite the substantial (expected) drop between 191 and 334 DOA, and our best guess is that the elevated level at 586 DOA represents the use of a mouse of a lower age by mistake. Gender differences were somewhat less obvious in that both manifested a prominent decline between 191 and 334 DOA—especially for GM1. The female showed a further substantial decrease at 586 DOA. 

### 3.2. Changes in Motor Function of Normal Mice with Aging

**Figure 4:**
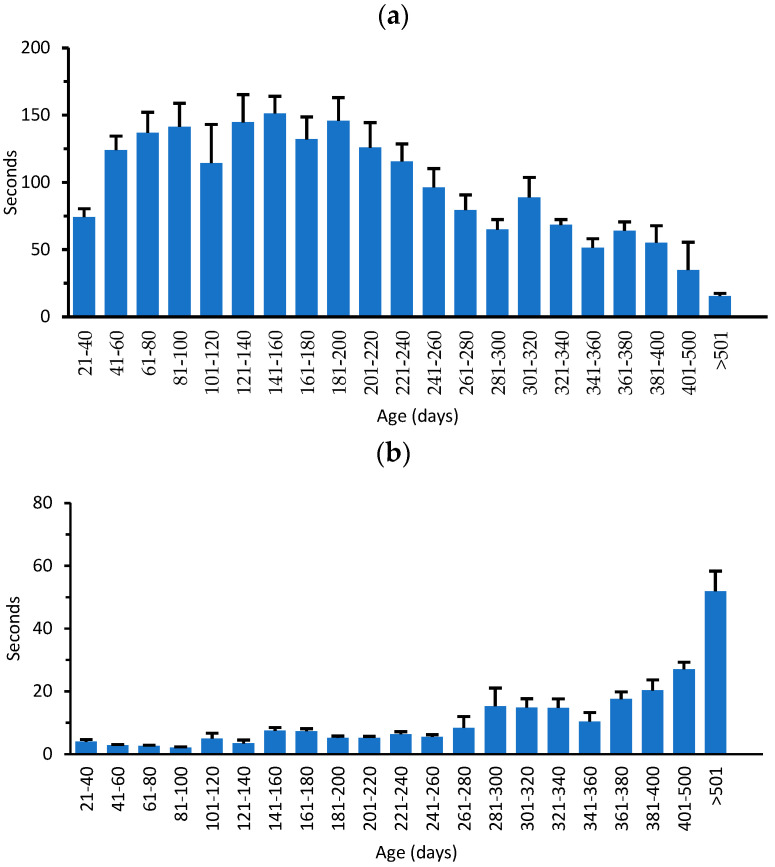
Motor changes with age: (**a**) motor coordination measured by grip duration test; (**b**) sensory-motor coordination measured by irritant removal test. Data indicate average ± SEM.

In order to demonstrate synchrony between ganglioside changes in peripheral tissues and motor function with aging, we carried out the grip duration and irritant removal tests used in previous studies [19]. Mice of both genders were employed from 21 to >501 DOA. Irritant removal (Figure 4b) remained optimal to approximately 260 DOA and declined thereafter (requiring more time for irritant removal) to >501 DOA.

### 3.3. Changes in the Memory Impairment of Normal Mice with Aging

**Figure 5:**
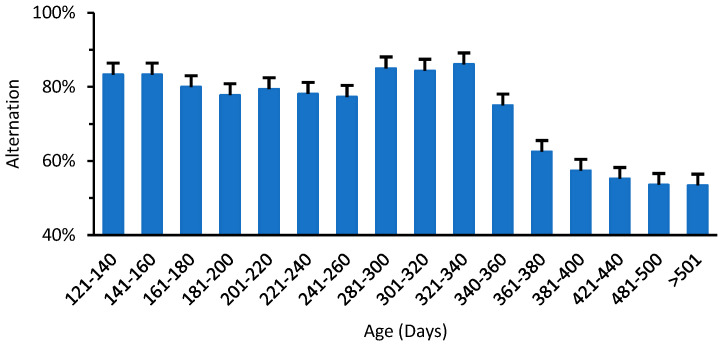
Percentage of spontaneous alternation rate in the T-maze test. Data indicate average ± SEM.

A similar synchrony was demonstrated between ganglioside changes and short-term memory, employing the T-maze method from previous studies [20]. Figure 5 shows the number of alternations of entries into the arms of the T-maze. Mice at 340 DOA showed a significant decrease in the number of entries into the preferred arm compared to younger mice. The memory to explore the nonaccessed arm with an alternation of almost 80% can be seen up to the age of approximately 340 days, though the decline in alternation % is steep beyond 361 DOA. It was thus noted that memory impairment started significantly later than motor impairment. 

## 4. Discussion

The proposal that PD patients suffer a significant deficiency in a-series gangliosides (both GM1 and GD1a) in the CNS was previously supported by the analysis of CNS tissues from the occipital cortex [21] and the nigrostriatal system [19,22] of PD patients. In those studies, evidence was reviewed and it was suggested that such a GM1 deficit in the CNS is the underlying cause of PD dysfunctions and pathologies due to the essentiality of GM1 in maintaining neuronal function and viability. Importantly, this deficiency occurred progressively, largely due to the aging process, as demonstrated by Svennerholm and coworkers for the a-series gangliosides [23,24]. However, insufficient attention has been accorded to this hypothesis concerning the PNS, despite the abundant evidence that such neurons suffer similar functional disruptions in PD. This aspect was alluded to in our recent demonstration of systemic deficiency of GM1/GD1a in PD [15], although that study did not demonstrate the progressivity of the deficit. We have attempted in the present study to provide such evidence, focusing on three peripheral tissues that are well recognized as pathological targets in PD. 

Following a previous demonstration by ourselves and others that such gangliosides progressively decline with age in the CNS of such mice [19,22,25], in this study, we have focused on the PNS in relation to the a-series ganglioside changes with age in normal mice. In those studies, the mouse brain was shown to be parallel to the human brain regarding the age-related decline in a-series gangliosides, and that, in turn, was correlated with several Parkinsonian pathologies that developed in the CNS of mice with subnormal GM1 [21,25]. We have shown that mice that are even partially deficient in the a-series gangliosides can also develop Parkinsonian symptoms in the periphery [20], thus raising the question of whether this too may be correlated with the age-related decline of the a-series. The present study has positively confirmed this, showing a substantial decrease in both GM1 and GD1a in three peripheral tissues from 191 days of age. The three tissues so studied, the colon, heart, and skin, were shown to be primary targets of PD, these symptoms often occurring as prodromal manifestations many years prior to the onset of movement dysfunctions [5]. We considered it informative to also include the age-related decline in behavior and memory of normal mice to show synchrony with the a-series decline in the aging process.

Our HPTLC study of the colon, one of the tissues used by Braak for his staging hypothesis [3], revealed for the males, a clear decline in both GM1 and GD1a between 191 and 334 DOA, and further decline at 586 days; female mice, on the other hand, showed a limited decline between 191 and 334 days but a more substantial decline at 586 days. Similar results were obtained for skin, an autonomic component of the PNS, which is often overlooked as being among those tissues manifesting prodromal non-motor symptoms of PD [26]. As pointed out in the latter study, seborrheic dermatitis is a principal symptom affecting the head, neck, upper trunk, and sternum areas. Tissue segments from the mouse heart, a major component of the autonomic nervous system that suffers an early loss of sympathetic innervation in PD [27,28], showed a similar a-series decline between 191 and 334 days, but in the males, an aberrant increase at 586 days. We do not know the cause of this departure from the expected decline (and the observed decline between 91 and 334 days) except to speculate a laboratory error (e.g., the use of a younger mouse by mistake). A limited colony size prevented the repetition of that experiment. 

Female mice showed a somewhat less age-related change in the a-series in all three tissues, which is consistent with the recent sex-related findings of Seyfried and coworkers that in contrast to the substantial loss of gangliosides in the substantia nigra of male PD subjects, no significant reductions were found in the substantia nigra of female PD subjects [29]. The risk of developing PD is twice as great in men as in women, although women have a higher mortality rate in addition to other PD-related disease manifestations. The present study suggests another sex-based PD difference concerning the age-based decline of a-series gangliosides in the PNS.

As to the cause of GM1 and GD1a deficiencies in PD, Svennerholm and coworkers showed this could be attributed in large part to the aging process itself, in contrast to the b-series gangliosides (GD1b, GT1b), which showed much less change over time in the normal brain [23]. This a-series decrease was most pronounced for GD1a [24], the well-recognized metabolic precursor to GM1; GD1a is known to serve as an effective reservoir for GM1 based on its co-localization with Neu3 neuraminidase, which converts GD1a to GM1 as the situation may require [30]. It was noted that age alone might not account for the total GM1 deficit in the PD brain; factors such as defective lysosomal hydrolases and other possible mechanisms possibly contribute [31]. However, the possibility that age and such additional mechanisms could also impinge on PNS neurons over time, has not, to our knowledge, been given similar consideration until now.

That such mechanisms do pertain to the PNS received tentative support in our recent demonstration of the systemic deficiency of GM1 and GD1a in PD-involved tissues in the periphery [15]. The present study extends those findings by demonstrating the contribution of aging to progressive a-series decline in the PNS, thus emphasizing the simultaneity between the CNS and PNS in this evolving neuropathology. Since this could not be performed with human peripheral tissues, we analyzed peripheral mouse tissues in accordance with the demonstration that the decline in a-series ganglioside in the mouse brain [19,25] progresses similarly to that in the human brain [23,24]. 

The essentiality of GM1 regarding neuronal function and viability relates to GM1 association with several proteins that require such association for effective functioning [32,33]. This has been well illustrated for aSyn, which binds GM1 with high affinity and specificity [34,35]. This association is necessary to retain aSyn in its alpha-helical, nonaggregating conformation. Supporting evidence for this mechanism was provided by an in vivo dispersion of aSyn aggregates by the GM1 application in rodents expressing such aggregates [19,36]. That GM1 and other gangliosides are essential for neuronal function was well illustrated in patients suffering disruption to the B4GALNT1 gene (GM2 synthase), which eliminates both a- and b-series gangliosides [37], and the even more devastating condition involving the disruption of the ST3GAL5 gene [38]. These two conditions occur infrequently and sporadically but are particularly prevalent in the Amish community, which also has the world’s highest incidence of PD [39].

Alpha-synuclein was initially localized to the presynaptic nerve terminal and the nucleus [40], although its functional role at neither locus has been fully clarified (and its role in the nucleus may be largely pathological). Several pathological reactivities have been identified for aSyn in the nucleus, an important one relating to GM1 being its downregulation of the Nurr1 transcription factor together with its downstream target Ret protein, the tyrosine hydroxylase component of the receptor for glial cell line-derived neurotrophic factor (GDNF); this is the neurotrophic factor (NTF) responsible for maintaining lifelong viability of catecholaminergic (e.g., dopaminergic) neurons [41]. Tropomyosin receptor kinase A (TrkA) and Tropomyosin receptor kinase B (TrkB) are two other NTF receptors that are tightly associated with GM1, pointing to GM1′s essential role in maintaining the lifelong viability of cortical neurons, the loss of which has been implicated in Alzheimer’s disease (AD) as well as late stages of PD. TrkA was shown to be significantly depleted in AD—approximately 50% in all regions except for the visual cortex [42], indicating a phenotypic downregulation of cholinergic function in AD. This points to the possibility of a systemic deficiency of GM1 in AD, as was shown for PD [15]. Another recent study demonstrated the anti-PD role of GM1 gangliosides resulting from the activation of autophagy-dependent α-Syn clearance [43].

A systemic deficiency of GM1 in PD patients, likely initiated and promoted by an age-related decline in the type demonstrated here, points to GM1 replacement therapy as a potential therapeutic solution to PD. This possibility received support in the clinical trials conducted by Dr. Jay Schneider and coworkers [44,45,46], whose therapeutic findings were apparently limited by the resistance of the blood–brain barrier to the passage of peripherally administered GM1 into the brain. Efforts are underway to solve that problem as well as methods to promote GM1 entry into neurons of the CNS and PNS—the neuronal membrane being another kind of barrier. There is a reason for optimism, in that these transport problems will be solved, allowing GM1 replacement to take its place as a disease-altering therapy for PD (and possibly for others, such as AD and Huntington’s disease) [47].

Finally, we would like to consider the question concerning the fact that despite the large number of individuals who experience a decline in a-series gangliosides (virtually all humans past a certain age), only a very small number develop PD. At least a partial explanation for this can be found in the finding of Svennerholm and coworkers [23,24], that individuals of the same age can manifest very different levels of the a-series. Thus, individuals starting life with GM1/GD1a in the very low-normal range might have an adequate level in their early years but in midlife could reach a point where their a-series falls below the threshold necessary to maintain full neuronal viability in the CNS and periphery. However, such speculation might not apply to all sporadic PD since additional factors besides aging might contribute to the suppression of the a-series. A prime candidate would be lysosomal dysfunction, which has been proposed to participate in the etiology and progression of PD and other neurodegenerative conditions [31,48]. The dysfunction of lysosomes could disrupt alpha-synuclein disposal, which would lead to its pathological accumulation with aggregation and Lewy body formation. Additional factors can be considered to have a role in a-series suppression [49]. In any case, the present study reveals the importance of considering the age-related decline in GM1 and GD1a in the periphery as potential contributors to peripheral symptoms of PD. 

## 5. Conclusions

The present study focused on the age-related decline in a-series gangliosides in the peripheral tissues of normal mice, especially those tissues most intimately affected by PD many years prior to the onset of movement dysfunctions. The results showed that normal aging alters GM1 and GD1a levels in the colon, skin, and heart tissues of mice, where a substantial decrease was observed between 191 and 586 DOA, the decline being slightly sharper in male mice compared to female mice. We propose that similar to the CNS, the decline in a-series gangliosides in the PNS of peripheral tissues contributes importantly to peripheral symptoms of PD.

## Data Availability

The data presented in this study are available on request from the corresponding author.

## Supplementary Materials

The following supporting information can be downloaded at: https://www.mdpi.com/article/10.3390/biomedicines11010209/s1, Figure S1: Depiction of a-series ganglioside in colon tissue of aging mice (a) HPTLC of male mice colon gangliosides (b) HPTLC of female mice colon gangliosides.; Figure S2: Depiction of a-series ganglioside in skin tissue of aging mice (a) HPTLC of male mice skin gangliosides; (b) HPTLC of female mice skin gangliosides.; Figure S3: Depiction of a-series ganglioside in heart tissue of aging mice (a) HPTLC of male mice heart tissue for detection of gangliosides; (b) HPTLC of female mice heart tissue for detection of GM1 and GD1a.
