# Supplementary material for: Age-Related Decline in Gangliosides GM1 and GD1a in Non-CNS Tissues of Normal Mice: Implications for Peripheral Symptoms of Parkinson’s Disease
Source: Biomedicines. 2023 Jan 14;11(1):209. doi: 10.3390/biomedicines11010209 (PMC9855670; doi:10.3390/biomedicines11010209)
Supplement: Supplementary file 1 [file biomedicines-11-00209-s001.zip › biomedicines-2071655-supplementary.pdf]

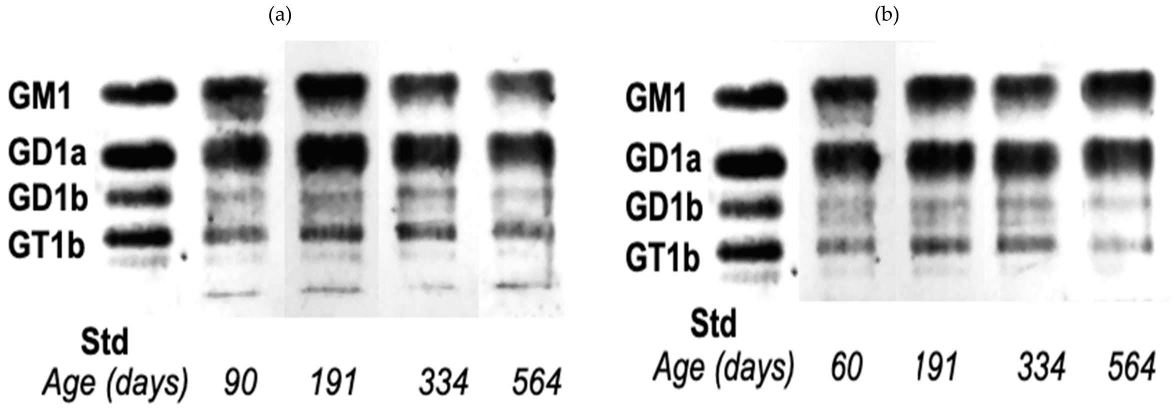

Figure S1. Depiction of a-series ganglioside in colon tissue of aging mice (a) HPTLC of male mice colon gangliosides (b) HPTLC of female mice colon gangliosides.

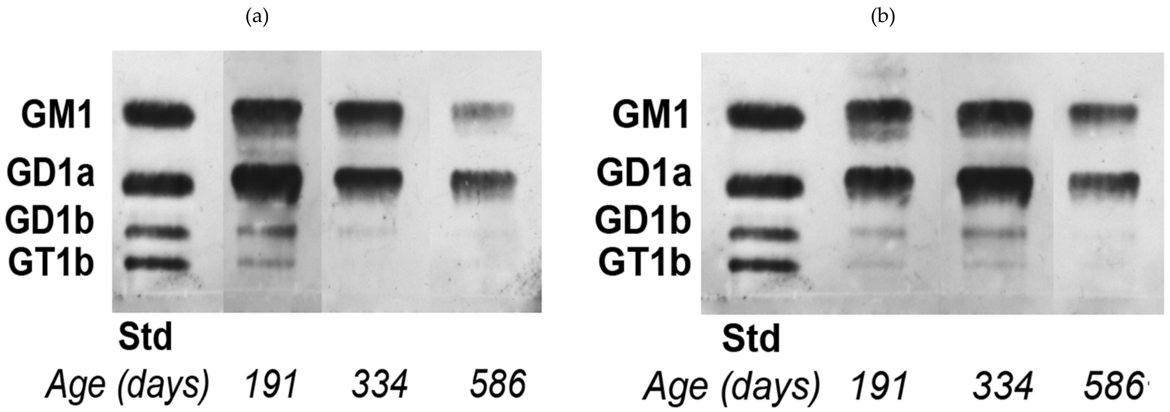

Figure S2. Depiction of a-series ganglioside in skin tissue of aging mice (a) HPTLC of male mice skin gangliosides; (b) HPTLC of female mice skin gangliosides.

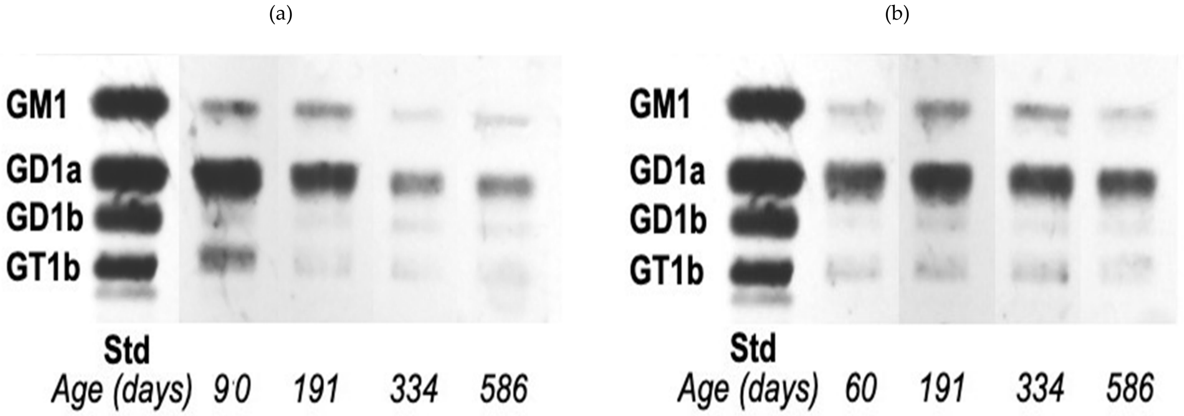

Figure S3. Depiction of a-series ganglioside in heart tissue of aging mice (a) HPTLC of male mice heart tissue for detection of gangliosides; (b) HPTLC of female mice heart tissue for detection of GM1 and GD1a.

These results concord with the results in the manuscript showing strong downward trend in a-series ganglioside with age in the peripheral system. It is particularly striking if we compare the intensity of a-series ganglioside between 191 and 586 DOA.
